# Bibliographical Notices

**Published:** 1839-01-05

**Authors:** 


### BIBLIOGRAPHICAL NOTICES.

*Jefferson Medical College. Professor* PATTISON'S *Introductory Lecture. Session* 1838–9. Philadelphia: published by order of the Committee. 1838. pp. 19.

AFTER a brief history of the progress of the Jefferson School, from its institution as a branch of a distant College, to its present condition with an independent charter, Prof. Pattison offers to the "consideration and example" of the medical student, sketches of the characters of two of the most distinguished lights of surgical science—Hunter and Physick. As a specimen of style and successful portraiture, we extract a descrption of the personal appearance of the American surgeon:

"The person of Dr. Physick was strongly indicative of his character. No man who was ever in his company could ever again forget his presence. His figure was rather under than over the common height; it was slight, but, on the whole, graceful. It was, however, on the 'temple of the soul,' in his noble head, where the strong delineation of his character was portrayed by Nature's chisel. His broad, expanded forehead, his aquiline nose, his compressed lips, and his round Grecian formed chin, appeared, from the pallid hue of his countenance, sculped in cold Parian marble; but the eye, full of thought, pensive, mild, and penetrating, shed the influences of life, energy, and feeling, over a countenance otherwise deathlike. For many years himself the victim of disease and suffering, and, from the nature of his pursuits, brought into hourly association in the chambers of death with scenes the most heart-rending to which our nature is subject, the subduing influences of melancholy had a saddening effect on the expression of his countenance and the tone of his character. But, although little given to cheerfulness, there was nothing of misanthropy or severity in his disposition. On the contrary, it was full of gentleness and tenderness. A sufferer himself, he sympathized most deeply, most sincerely, with the sufferer, and devoted his whole life and the whole energies of his mind to the mitigation of the pains and miseries of mortality."

---

I.—*Some Account of the Typhus Exanthematicus, as observed in St. Bartholomew's Hospital, London, in* 1837–38. By CHARLES WEST, M. D., Graduate in Medicine and Surgery in the University of Berlin.

II.—*A Statistical Inquiry on Fever, being an attempt to ascertain the Prevalence, Susceptibility, Intensity, and Prognosis, with some Observations on the Influence of Medical Treatment.* By AUTHUR SAUNDERS THOMSON, M. D., &c.

IN the Edinburgh Medical and Surgical Journal, for July, 1838, we find two interesting articles on Typhus Fever. The paper by Dr. West is an account of the symptoms, pathology, and treatment of the disease as it appeared in the last epidemic at London; that of Dr. Thomson is a statistical statement of the results obtained from comparing the practice of various hospitals in Great Britain and Ireland. Of course, the two papers present totally distinct points of interest. That of Dr. West should be first examined, as our first object in all such cases is to identify the disease before we proceed to inquire into its rate of mortality and relative frequency of appearance.

First, of the appearances found after death by Dr. West. These were obtained from the dissection of ten bodies—a small number to yield accurate results, more especially as the appearances on dissection are not related in a very careful manner.

It appears that in no case was there much morbid alteration of the glands of Peyer; in one only they "appeared enlarged." In other cases there was no morbid appearance whatever in the alimentary canal. In five cases there were increased vascularity and softening of the whole, or of a portion of the intestinal canal or stomach. The liver was always healthy, but the spleen was enlarged and softened in six cases.

We may, therefore, conclude that, as far as the abdominal organs are concerned, the disease was typhus, and not typhoid fever, which latter term we use as synonymous with dothinenteritis.

The appearances of the brain were various. In nine cases the membranes were more or less congested. There was serous effusion into the arachnoid in six cases; once there was some softening of the cerebellum, and of the left anterior lobe of tbe cerebrum.

"The respiratory organs were only once found in a healthy condition, and that one case we can scarcely reckon with the others, it being that of the patient who died on the forty-third day." These lesions were chiefly extreme congestion of the bronchial mucous membrane, extending in many cases to the lower lobes of the lung. The heart, it is said, presented no peculiar morbid appearance, and the state of the blood is not mentioned.

From this review of the pathological phenomena of the fever described by Dr. West, we may conclude that it is identical in its anatomical lesions with the epidemic of typhus which occurred in Philadelphia in the years 1836 and 1837, and ceased in the early part of 1838. This epidemic was chiefly local, and confined to a portion of the town. It was described by Dr. Gerhard, in two papers published in the American Journal of Medical Sciences, (February and August, 1837.)

The symptoms related by Dr. West are equally conclusive, that the disease was the same. The action of the bowels was not disturbed in the great majority of cases; diarrhœa occurred only in one-sixth, nor did it last longer than forty-eight or sixty hours, in more than four patients. The evacuations were watery and yellow. There was pain of the abdomen in thirty cases, but in nine only was it severe. The bowels were constipated in thirteen cases, and there was tympanitic distension of the abdomen in eleven.

The state of the tongue was variable, it became dry and fuliginous when the brain was much affected. This observation is usually made in this variety of fever.

The nervous symptoms are far more important than those connected with the digestive canal. Pain in the head was always found when the patients were admitted before the occurrence of stupor or delirium. It came on at the beginning of the disease, either before or closely after the first shivering; it could not be discovered when there was either delirium or stupor, and was, therefore, most frequent in the first eight or ten days. There was more or less delirium in thirty-three cases, including all those which were fatal; it lasted on the average seven days, and was extremely various in its character,—and, in the slighter cases, was observed only during the night. It is stated that tremor or subsultus was frequent; very probably it was always present. The strength was extremely prostrated, even in the stoutest individuals, and the symptoms of disordered hearing and sight were very frequent. The suffusion of the eye, which is so characteristic of this disease, was most marked at the commencement; the eye became dull and vacant on the approach of death. There was some affection of the respiratory organs in twenty-one cases. A crepitant rhonchus was heard in the lungs of ten patients, four of whom died; in the others the signs were chiefly those of bronchitis.

The affections of the circulatory organs are very imperfectly described by Dr. West; the pulse was on the average one hundred and twenty. The undulatory character of the pulse, which is so frequent in these forms of fever, was commonly met with.

The symptoms afforded by the surface of the body are much morecar efully noted. Forty-two cases presented the peculiar measle-like eruption described by so many authors, "which first showed itself from the sixth to the eighth day." "Of the eighteen cases in which no eruption was observed, five only were admitted before the eighth day of the disease; it is, therefore, very probable that the eruption had existed in some of these patients, but had disappeared before their admission." These spots would fade within two or three days, but would not entirely disappear for a long time afterwards, often for a fortnight.

The appearance of this eruption was identical with that of the fever observed at Philadelphia, a few years since, while it is totally unlike the rose coloured spots of typhoid fever, which are almost entirely confined to the abdomen, and are much less numerous than the measle-like eruption of the typhus. The eruption would sometimes become dark, and resemble the ordinary petechiæ, which was also a matter of common remark at Philadelphia.

Amongst the other symptoms of the exterior, Dr. West particularly notices the burning pungent heat of the surface, which left a disagreeable sensation upon the fingers for some time after the hand was removed from the body. He does not mention whether the exhalation from the skin was characterized by the disagreeable pungent odour which was so frequently observed in the epidemic at Philadelphia.

The mortality was one in four, two-sevenths, showing that the epidemic was extremely severe. The mortality varies so much in the epidemics of typhus, that practitioners who are not aware of the circumstance would be led into egregious errors. The mortality at the hospital, when the greatest number of cases occurred at Philadelphia, was enormous—about one in three cases, that is, including a very large proportion who died immediately after admission: these patients, for the most part, had received no medical aid whatever previously to their admission, and entered very late in the disease. They were to be found amongst the most miserable classes of the population. At the very same hospital, another epidemic, which was chiefly confined to passengers arrived in the emigrant ships from Europe, offered a very small ratio of mortality, scarcely three or four per cent.

In proportion as the mortality of an epidemic of typhus is great, the contagion is more active. Thus, in the epidemic at St. Bartholomew's hospital, Dr. West states that "eleven gentlemen, who were in the habit of frequenting the hospital, have been attacked by the fever, to which nine had fallen victims. Sixteen nurses, and twenty-one patients admitted for other affections, have likewise suffered from the disease, which terminated fatally in ten instances; and I do not doubt, but that many similar cases occurred which did not come under my notice." The epidemic of 1836–7, at Philadelphia, offered the contagious form of the disease in a very high degree; and here, as well as at London, the fever seemed most severe and most contagious during the coldest months of the year.

The treatment is not stated with sufficient detail, nor is it analysed with sufficient care, to enable us to draw any very accurate conclusions from it. The disease was never cut short after chill had occurred,—that is, after it had fairly set in. The observations made in this city, as well as in all other places, confirm the statement of Dr. West. Nevertheless, much may always be done; and the disease may be so far mitigated, as to prevent the developement of local and secondary affections.

Such of our readers as are familiar with the subject of typhus fever, will regard with much interest the analysis which we have just given of Dr. West's paper. The very decided distinction between the typhus of Great Britain and Ireland, and the typhoid fever which is described by the late French writers, is not even yet understood by many practitioners. Hence, in reading the works of Louis and Chomel on the one hand, and of the British physicians on the other, we must not forget that we are studying two diseases which differ from each other in many important respects. Our present object is not to draw a parallel between them, but simply to present such an analysis of Dr. West's paper as will show what the disease is. We may afterwards return to this subject, and will give a more connected account of the two diseases.

The paper of Dr. Thomson contains numerous statistical facts relative to fever,—that is, to the typhus of Great Britain—the typhoid being there of much more rare occurrence. We shall extract from it in the next number the most interesting data.

---

Sidney A. Doane, M. D., has been elected Professor of Physiology in the University of New York.

